# Supplementary material for: Modeling glioblastoma heterogeneity as a dynamic network of cell states
Source: Mol Syst Biol. 2021 Sep 16;17(9):e10105. doi: 10.15252/msb.202010105 (PMC8444284; doi:10.15252/msb.202010105)
Supplement: Supplementary file 5 — Source Data for Figure 3 [file MSB-17-e10105-s001.zip › Figure3A_sourcedata/GSEA_3065/hallmarks_state2.GseaPreranked.1623416269049/HALLMARK_INFLAMMATORY_RESPONSE.html]

Details for gene set HALLMARK\_INFLAMMATORY\_RESPONSE[GSEA]

|  || Dataset | state2 |
| Phenotype | NoPhenotypeAvailable |
| Upregulated in class | na\_neg |
| GeneSet | HALLMARK\_INFLAMMATORY\_RESPONSE |
| Enrichment Score (ES) | -0.56427574 |
| Normalized Enrichment Score (NES) | -2.0157795 |
| Nominal p-value | 0.0 |
| FDR q-value | 0.0 |
| FWER p-Value | 0.0 |
Table: GSEA Results Summary

  

Fig 1: Enrichment plot: HALLMARK\_INFLAMMATORY\_RESPONSE      
 Profile of the Running ES Score & Positions of GeneSet Members on the Rank Ordered List

  

| PROBE | GENE SYMBOL | GENE\_TITLE | RANK IN GENE LIST | RANK METRIC SCORE | RUNNING ES | CORE ENRICHMENT || 1 | SRI |  |  | 181 | 0.293 | 0.0265 | No |
| 2 | ADRM1 |  |  | 508 | 0.179 | 0.0198 | No |
| 3 | IFNGR2 |  |  | 1538 | 0.085 | -0.0768 | No |
| 4 | RELA |  |  | 1694 | 0.077 | -0.0812 | No |
| 5 | RHOG |  |  | 1944 | 0.067 | -0.0973 | No |
| 6 | RIPK2 |  |  | 1947 | 0.067 | -0.0871 | No |
| 7 | HIF1A |  |  | 2182 | 0.058 | -0.1031 | No |
| 8 | EIF2AK2 |  |  | 2212 | 0.057 | -0.0973 | No |
| 9 | LY6E |  |  | 2588 | 0.045 | -0.1302 | No |
| 10 | TNFAIP6 |  |  | 2720 | 0.041 | -0.1378 | No |
| 11 | SLC7A1 |  |  | 3014 | 0.033 | -0.1638 | No |
| 12 | GNAI3 |  |  | 3356 | 0.027 | -0.1960 | No |
| 13 | RAF1 |  |  | 3447 | 0.025 | -0.2017 | No |
| 14 | NMI |  |  | 3492 | 0.024 | -0.2027 | No |
| 15 | MYC |  |  | 3842 | 0.018 | -0.2371 | No |
| 16 | HAS2 |  |  | 4545 | 0.007 | -0.3110 | No |
| 17 | NAMPT |  |  | 4573 | 0.006 | -0.3129 | No |
| 18 | SCN1B |  |  | 5213 | -0.004 | -0.3805 | No |
| 19 | NFKB1 |  |  | 5273 | -0.004 | -0.3861 | No |
| 20 | HRH1 |  |  | 5451 | -0.008 | -0.4038 | No |
| 21 | PDE4B |  |  | 5498 | -0.008 | -0.4074 | No |
| 22 | PVR |  |  | 6162 | -0.019 | -0.4751 | No |
| 23 | NFKBIA |  |  | 6199 | -0.020 | -0.4759 | No |
| 24 | SEMA4D |  |  | 6307 | -0.022 | -0.4839 | No |
| 25 | PDPN |  |  | 6446 | -0.024 | -0.4948 | No |
| 26 | SLC31A1 |  |  | 6496 | -0.025 | -0.4961 | No |
| 27 | ADORA2B |  |  | 6618 | -0.028 | -0.5047 | No |
| 28 | IRF7 |  |  | 6755 | -0.031 | -0.5144 | No |
| 29 | ABI1 |  |  | 6766 | -0.031 | -0.5106 | No |
| 30 | PLAUR |  |  | 6906 | -0.034 | -0.5201 | No |
| 31 | KLF6 |  |  | 7010 | -0.037 | -0.5253 | No |
| 32 | KIF1B |  |  | 7160 | -0.041 | -0.5348 | No |
| 33 | GABBR1 |  |  | 7437 | -0.048 | -0.5567 | Yes |
| 34 | RGS16 |  |  | 7474 | -0.050 | -0.5528 | Yes |
| 35 | KCNJ2 |  |  | 7552 | -0.052 | -0.5529 | Yes |
| 36 | IRF1 |  |  | 7563 | -0.052 | -0.5458 | Yes |
| 37 | CX3CL1 |  |  | 7596 | -0.053 | -0.5409 | Yes |
| 38 | F3 |  |  | 7662 | -0.055 | -0.5393 | Yes |
| 39 | IFNAR1 |  |  | 7683 | -0.056 | -0.5327 | Yes |
| 40 | PSEN1 |  |  | 7761 | -0.058 | -0.5317 | Yes |
| 41 | ACVR2A |  |  | 7795 | -0.059 | -0.5260 | Yes |
| 42 | OSMR |  |  | 7864 | -0.062 | -0.5236 | Yes |
| 43 | SLC11A2 |  |  | 8006 | -0.068 | -0.5280 | Yes |
| 44 | NDP |  |  | 8125 | -0.073 | -0.5293 | Yes |
| 45 | CD82 |  |  | 8151 | -0.074 | -0.5203 | Yes |
| 46 | ITGA5 |  |  | 8155 | -0.075 | -0.5089 | Yes |
| 47 | SLC4A4 |  |  | 8359 | -0.086 | -0.5171 | Yes |
| 48 | AHR |  |  | 8513 | -0.097 | -0.5182 | Yes |
| 49 | CSF1 |  |  | 8560 | -0.101 | -0.5074 | Yes |
| 50 | ATP2B1 |  |  | 8584 | -0.102 | -0.4940 | Yes |
| 51 | ABCA1 |  |  | 8635 | -0.106 | -0.4828 | Yes |
| 52 | CHST2 |  |  | 8670 | -0.109 | -0.4694 | Yes |
| 53 | P2RX7 |  |  | 8760 | -0.117 | -0.4605 | Yes |
| 54 | MET |  |  | 8800 | -0.122 | -0.4456 | Yes |
| 55 | TPBG |  |  | 8829 | -0.127 | -0.4288 | Yes |
| 56 | PTPRE |  |  | 8861 | -0.131 | -0.4116 | Yes |
| 57 | ATP2C1 |  |  | 8879 | -0.133 | -0.3925 | Yes |
| 58 | BST2 |  |  | 8903 | -0.136 | -0.3737 | Yes |
| 59 | EMP3 |  |  | 8918 | -0.137 | -0.3537 | Yes |
| 60 | ATP2A2 |  |  | 8946 | -0.141 | -0.3346 | Yes |
| 61 | TAPBP |  |  | 8966 | -0.144 | -0.3141 | Yes |
| 62 | AXL |  |  | 9023 | -0.152 | -0.2962 | Yes |
| 63 | LDLR |  |  | 9117 | -0.170 | -0.2795 | Yes |
| 64 | SELENOS |  |  | 9135 | -0.174 | -0.2541 | Yes |
| 65 | CALCRL |  |  | 9236 | -0.205 | -0.2326 | Yes |
| 66 | BTG2 |  |  | 9239 | -0.207 | -0.2005 | Yes |
| 67 | CDKN1A |  |  | 9301 | -0.230 | -0.1710 | Yes |
| 68 | ITGB8 |  |  | 9362 | -0.294 | -0.1315 | Yes |
| 69 | TIMP1 |  |  | 9387 | -0.334 | -0.0817 | Yes |
| 70 | DCBLD2 |  |  | 9431 | -0.558 | 0.0010 | Yes |
Table: GSEA details [plain text format]

  

Fig 2: HALLMARK\_INFLAMMATORY\_RESPONSE: Random ES distribution      
 Gene set null distribution of ES for **HALLMARK\_INFLAMMATORY\_RESPONSE**
